# Genomic Epidemiology and Active Surveillance to Investigate Outbreaks of Hantaviruses

**DOI:** 10.3389/fcimb.2020.532388

**Published:** 2021-01-08

**Authors:** Won-Keun Kim, Seungchan Cho, Seung-Ho Lee, Jin Sun No, Geum-Young Lee, Kyungmin Park, Daesang Lee, Seong Tae Jeong, Jin-Won Song

**Affiliations:** ^1^ Department of Microbiology, College of Medicine, Hallym University, Chuncheon, South Korea; ^2^ Institute of Medical Science, College of Medicine, Hallym University, Chuncheon, South Korea; ^3^ Department of Microbiology, Korea University College of Medicine, Seoul, South Korea; ^4^ BK21 Graduate Program, Department of Biomedical Sciences, Korea University College of Medicine, Seoul, South Korea; ^5^ 4th R&D Institute, Agency for Defense Development, Daejeon, South Korea

**Keywords:** RNA viruses, tracking hantaviral genomes, next-generation sequencing, epidemiological survey, rodent trapping, preventive strategies

## Abstract

Emerging and re-emerging RNA viruses pose significant public health, economic, and societal burdens. Hantaviruses (genus *Orthohantavirus*, family *Hantaviridae*, order *Bunyavirales*) are enveloped, negative-sense, single-stranded, tripartite RNA viruses that are emerging zoonotic pathogens harbored by small mammals such as rodents, bats, moles, and shrews. Orthohantavirus infections cause hemorrhagic fever with renal syndrome (HFRS) and hantavirus cardiopulmonary syndrome in humans (HCPS). Active targeted surveillance has elucidated high-resolution phylogeographic relationships between patient- and rodent-derived orthohantavirus genome sequences and identified the infection source by temporally and spatially tracking viral genomes. Active surveillance of patients with HFRS entails 1) recovering whole-genome sequences of Hantaan virus (HTNV) using amplicon (multiplex PCR-based) next-generation sequencing, 2) tracing the putative infection site of a patient by administering an epidemiological questionnaire, and 3) collecting HTNV-positive rodents using targeted rodent trapping. Moreover, viral genome tracking has been recently performed to rapidly and precisely characterize an outbreak from the emerging virus. Here, we reviewed genomic epidemiological and active surveillance data for determining the emergence of zoonotic RNA viruses based on viral genomic sequences obtained from patients and natural reservoirs. This review highlights the recent studies on tracking viral genomes for identifying and characterizing emerging viral outbreaks worldwide. We believe that active surveillance is an effective method for identifying rodent-borne orthohantavirus infection sites, and this report provides insights into disease mitigation and preparedness for managing emerging viral outbreaks.

## Introduction

Hantaviruses (genus *Orthohantavirus*) are enveloped, negative-sense, single-stranded RNA viruses belonging to the family *Hantaviridae* and order *Bunyavirales* (Abudurexiti et al., 2019). The orthohantavirus genome consists of tripartite RNA segments (large, medium, and small) encoding an RNA-dependent RNA polymerase (RdRp), two surface glycoproteins (Gn and Gc), and a nucleocapsid protein (N) (Vaheri et al., 2013). Hantaviruses are emerging zoonotic pathogens harbored by small mammal hosts such as rodents, bats, moles, and shrews (Witkowski et al., 2016). Hantavirus transmission to humans occurs when a person inhales aerosols or dust particles of orthohantavirus-contaminated rodent urine, feces, or saliva. Personnel engaged in forestry and farming practices in an area with a large rodent population are particularly vulnerable to these viruses (Forbes et al., 2018). Hantaviruses cause two types of diseases in humans, whereas infected rodents are asymptomatic. Hemorrhagic fever with renal syndrome (HFRS) is mainly caused by Hantaan virus (HTNV), Seoul virus, Dobrava-Belgrade virus, and Puumala virus (PUUV) **(** **Table 1** **)**. In the Americas, hantavirus cardiopulmonary syndrome (HCPS) is caused by Sin Nombre virus and Andes virus. Approximately 150,000 cases of HFRS are reported annually, with a case fatality rate (CFR) of <1–12% (Jonsson et al., 2010). The clinical course of HFRS is primarily characterized by fever, circulatory collapse with hypotension, hemorrhage, and acute kidney injury (AKI) (Jiang et al., 2016). Annually, approximately 200 cases of HCPS are reported with a CFR of up to 40% (Macneil et al., 2011). HCPS is characterized by fever, headache, malaise, myalgia, dyspnea, cough, and gastrointestinal complaints such as nausea, vomiting, and diarrhea (Chang et al., 2007). PUUV is responsible for thousands of nephropathia epidemica (NE) cases per year in Europe. NE is a milder form of HFRS that also includes thrombocytopenia and AKI (Jiang et al., 2017). Although inactivated orthohantavirus vaccines are developed from the brain cells of suckling mice or cell cultures, effective therapies are currently unavailable (Cho and Howard, 1999; Yi et al., 2018).

**Table 1:** Hantaviruses causing hemorrhagic fever with renal syndrome (HFRS) and hantavirus cardiopulmonary syndrome (HCPS).

Virus species	Abbreviation	Disease type	Rodent host	Geographic distribution	Reference
*Andes orthohantavirus*	Andes virus (ANDV)	HCPS	*Oligoryzomys longicaudatus*	South America	(Lopez et al., 1996)
	Castelo dos Sonhos virus (CASV)	HCPS	*Oligoryzomys eliurus*	South America	(Johnson et al., 1999)
	Lechiguanas virus (LECV = LECHV)	HCPS	*Oligoryzomys flavescens*	South America	(Levis et al., 1997)
	Orán virus (ORNV)	HCPS	*Oligoryzomys longicaudatus*	South America	(Lopez et al., 1996)
*Bayou orthohantavirus*	Bayou virus (BAYV)	HCPS	*Oryzomys palustris*	North America	(Morzunov et al., 1995)
	Catacamas virus (CATV)	HCPS	*Oligoryzomys couesi*	Central America	(Milazzo et al., 2006)
*Black Creek Canal orthohantavirus*	Black Creek Canal virus (BCCV)	HCPS	*Sigmodon hispidus*	North America	(Rollin et al., 1995)
*Choclo orthohantavirus*	Choclo virus (CHOV)	HCPS	*Oligoryzomys fulvescens*	North America	(Vincent et al., 2000)
*Dobrava-Belgrade orthohantavirus*	Dobrava virus (DOBV)	HFRS	*Apodemus flavicollis*	Europe	(Avsic-Zupanc et al., 1995)
	Kurkino virus (KURV)	HFRS	*Apodemus agrarius*	Europe	(Plyusnin et al., 1999b)
	Saaremaa virus (SAAV)	HFRS/NE	*Apodemus agrarius*	Europe	(Nemirov et al., 1999)
	Sochi virus (SOCV)	HFRS	*Apodemus ponticus*	Europe	(Klempa et al., 2008)
*El Moro Canyon orthohantavirus*	El Moro Canyon virus (ELMCV)	HCPS	*Reithrodontomys megalotis*, *Reithrodontomys sumichrasti*	North America	(Hjelle et al., 1994)
*Hantaan orthohantavirus*	Hantaan virus (HTNV)	HFRS	*Apodemus agrarius*	Russia, Asia	(Lee et al., 1978)
	Amur virus (AMRV)	HFRS	*Apodemus peninsulae*	Russia, Asia	(Lokugamage et al., 2004)
	Soochong virus (SOOV)	HFRS	*Apodemus peninsulae*	Asia	(Baek et al., 2006)
*Laguna Negra orthohantavirus*	Laguna Negra virus (LANV)	HCPS	*Calomys callosus*	South America	(Johnson et al., 1997)
	Maripa virus (MARV)	HCPS	*Unknown*	South America	(Matheus et al., 2012)
	Río Mamoré virus (RIOMV)	HCPS	*Oligoryzomys microtis*	South America	(Bharadwaj et al., 1997)
*Luxi orthohantavirus*	Lúxī virus (LUXV)	HFRS	*Eothenomys miletus*	Asia	(Zhang et al., 2011)
*Puumala orthohantavirus*	Puumala virus (PUUV)	HFRS/NE	*Myodes glareolus*	Europe, Asia	(Brummer-Korvenkontio et al., 1980)
	Muju virus (MUJV)	HFRS	*Myodes regulus*	Asia	(Song et al., 2007)
*Sangassou orthohantavirus*	Sangassou virus (SANGV)	HFRS	*Hylomyscus simus*	Africa	(Klempa et al., 2012)
*Seoul orthohantavirus*	Seoul virus (SEOV)	HFRS	*Rattus norvegicus* *Rattus rattus*	Worldwide	(Lee et al., 1982)
	gōu virus (GOUV)	HFRS	*Rattus norvegicus, Rattus rattus, Rattus tanezumi*	Asia	(Wang et al., 2000)
*Sin Nombre orthohantavirus*	Sin Nombre virus (SNV)	HCPS	*Peromyscus maniculatus*	North America	(Nichol et al., 1993)
	New York virus (NYV)	HCPS	*Peromyscus leucopus*	North America	(Song et al., 1994)
*Thailand orthohantavirus*	Thailand virus (THAIV)	HFRS	*Bandicota indica*	Asia	(Xiao et al., 1994)
*Tula orthohantavirus*	Tula virus (TULV)	HFRS	*Microtus arvalis* *Microtus rossiaemeridionalis*	Europe	(Plyusnin et al., 1994)
	Adler virus (ADLV)	HFRS	*Microtus majori*	Europe	(Tkachenko et al., 2015)

HFRS, Hemorrhagic fever with renal syndrome; HCPS, Hantavirus cardiopulmonary syndrome; NE, Nephropathia epidemica.

**Figure 1:**
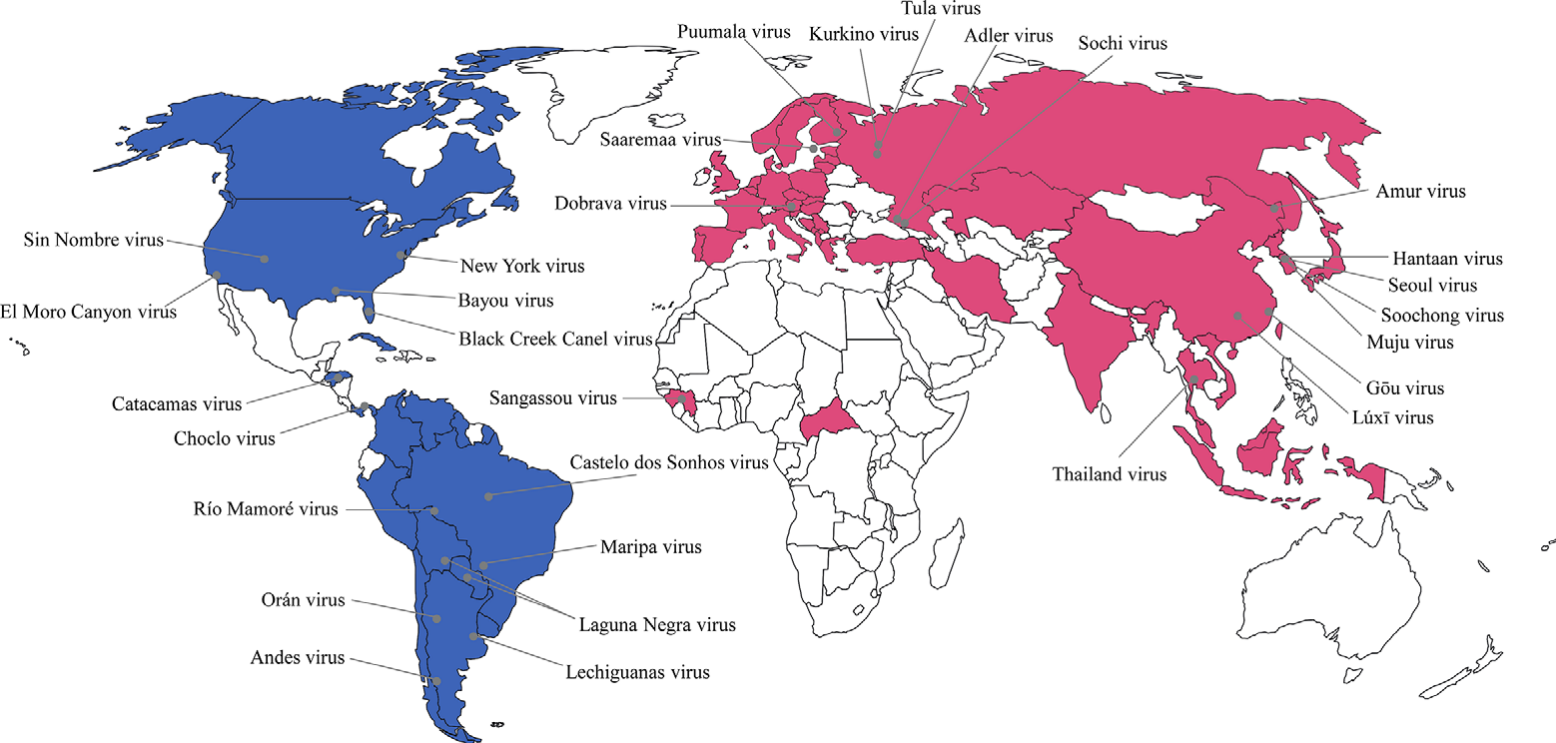
Global geographic distribution of hantaviruses as etiological agents of hemorrhagic fever with renal syndrome (HFRS) and hantavirus cardiopulmonary syndrome (HCPS) in humans. The representative hantaviral disease in each continent was marked as different color. Pink color indicates HFRS and blue color indicates HCPS. The gray circle represents the rodent-borne hantaviruses first characterized in nature.

Orthohantavirus-induced diseases pose a public health threat worldwide owing to significant morbidity and mortality rates **(** **Figure 1** **)**. Robust epidemiological investigation and surveillance are crucial for timely response to orthohantavirus outbreaks. Conventional epidemiological measures such as obtaining clinical data, determining case definitions, and contact tracing are necessary for understanding virus dynamics. However, these measures may be difficult to undertake in many cases, and detailed assessments may be required to support effective interventions. To address these challenges, viral genomic data are being increasingly generated to complement epidemiological data (Pybus et al., 2015; Ladner et al., 2019).

In this review, we summarize active targeted surveillance developed for emerging hantaviruses by focusing on next-generation sequencing (NGS), epidemiological interviews, and targeted rodent trapping. This includes 1) genome tracking using whole-genome sequencing, 2) follow-up of suspected infection sites using patient interview data, and 3) rodent trapping to capture the natural reservoir host. Additionally, this review presents effective preventive and mitigation strategies for rodent-borne orthohantavirus outbreaks using active surveillance.

## Generating Genomic Epidemiological Data Using Partial Viral Genome Sequences

Viral genome sequences have been increasingly used for inferring genotypes or strain-to-strain analyses during an outbreak. The tracking of emerging viruses initially relied on analyzing partial genome sequences to characterize the infection source, epidemic strains, and spreading patterns. However, studies investigating genomic epidemiology by analyzing partial genomic sequences are limited. Typically, partial genome sequences are studied to surveil the emergence of HTNV, and continued rodent trapping in endemic areas of the Republic of Korea (ROK) are employed (Song et al., 2000; Sames et al., 2009; Klein et al., 2011; Klein et al., 2012; Klein et al., 2015; Kim et al., 2017). In 2005, four US soldiers developed HFRS at training sites near the Demilitarized Zone, ROK (Song et al., 2009). The partial M segment of HTNV was confirmed in serum samples of the patients, and the lung tissues of *Apodemus agrarius* were collected from the trapping sites. The partial genomic sequence of HTNV showed a phylogeographic relationship between US soldiers diagnosed with HFRS and HTNV-positive *A. agrarius*. The potential rodent exposure sites in HCPS cases in the United States were identified *via* patient interviews (Hjelle et al., 1996). Partial viral genome sequences obtained from rodent lung tissues sampled from potential exposure sites showed homologous genetic lineages between patient- and rodent-derived viral sequences. Six out of nine Finnish patients with NE were diagnosed positive for either S or M segments of PUUV (Plyusnin et al., 1999a). Partial viral genome sequence analysis showed that these patients shared a common ancestor with previously reported Finnish bank voles or patients with NE infected with PUUV strains. The comparative genomics showed that the partial M segment sequence of human PUUV strain was identical to the corresponding sequence obtained from bank voles at the original infection site. In Germany, out of the 31 HFRS cases, 3 were diagnosed positive for the partial S segment of PUUV (Schilling et al., 2007). Small mammals at the potential exposure site were captured for tracing the source of the infection in HFRS patients. The partial S segment sequence analysis showed the presence of a phylogenetic relationship between the three patients and bank voles, indicating that bank voles were the source of the infection. However, the reverse transcription polymerase chain reaction (RT-PCR) technique has its limitations for analyzing the viral genome sequences in clinical specimens only during the viremic period (Bi et al., 2008). In addition, partial genome sequences may not always reflect the precise phylogenetic position, which has to be confirmed by performing whole-genome sequence analysis. Genomic variants including highly variable genomic mutations, reassortment, and recombination limit the application of partial genome sequences (Andersen et al., 2015).

## Complete Viral Genome Sequences for Genomic Epidemiology for Using Next-Generation Sequencing

To investigate genomic epidemiology, whole-genome sequencing elicits precise phylogeographic analysis because of the detection of genetic variants (Klempa, 2018). In Finland, a complete PUUV genomic sequence obtained from a critical HFRS case was compared with viral genome sequences obtained from local bank voles (Plyusnina et al., 2012). After the discovery of NGS technology, whole-genome sequences have been extensively used for “viral” genomic epidemiology from human and natural reservoir specimens. Tracking of the whole-genome sequences of Ebola virus (EBOV) from patients revealed human-to-human transmission of the virus in Guinea in 2013 (Baize et al., 2014; Gire et al., 2014; Carroll et al., 2015). This genomic epidemiology corroborated the notion that proactive real-time viral genome sequencing and human population surveillance represent simple and cost-effective methods of mitigating viral spreads in locations that are most vulnerable to infectious diseases. Phylodynamic analyses (including haplotype network generation, root-to-tip divergence calculation, and expected sampling time estimation) describe the factors driving virus spread and outbreak (Grubaugh et al., 2019). These analyses played a critical role in understanding Ebola flare-ups and identifying persistently infected and surviving patients with EBOV, thereby identifying sexual transmission of EBOV (Mate et al., 2015; Blackley et al., 2016). Lassa virus (LASV) and yellow fever virus outbreaks occurred in Nigeria in 2018. Researchers at the African Center of Excellence for Genomics of Infectious Diseases (Redeemer’s University, Ede, Nigeria) successfully guided outbreak responses in the country using real-time NGS (Siddle et al., 2018; Ajogbasile et al., 2019). Viral whole-genome sequence analyses showed that LASV spreads *via* repeated transmission from local rodent reservoirs rather than sustained human-to-human transmission. In 2016, genomic surveillance and phylodynamic analyses of Zika virus (ZIKV) genomes from infected patients and *Aedes aegypti* mosquitoes showed that the Caribbean Islands were the main source of the local ZIKV outbreak in Florida, whereas multiple ZIKV transmissions occurred throughout the Americas (Grubaugh et al., 2017). Multiplex PCR-based NGS enabled whole-genome sequencing of HTNV from human and rodent specimens collected in endemic and military training areas (Kim et al., 2016). Comparative genomic analysis of viral genome sequences from HFRS patients and rodents demonstrated phylogeographic relationships between patients with HFRS and HTNV-positive *A. agrarius* captured at suspected patient infection sites. Viral genome sequencing can be scaled up to match the evolution timescale, and these analytical techniques are increasingly exploited for timely communication of information to respond to an outbreak.

However, whole-genome sequencing directly from clinical and environmental specimens has some limitations. Various NGS methods have been developed to overcome the ultralow virus concentrations. A recent study reported the use of effective approaches for sequencing viral genomes by comparing different target enrichment NGS methods (No et al., 2019). Multiplex PCR-based NGS (amplicon sequencing) exhibited high coverage rates of HTNV in *A. agrarius* lung tissues without cultivating the infectious viral particles. However, the experimental techniques and materials used for NGS preparation are at a potential risk of contamination (Laurence et al., 2014). Contamination may cause difficulty in characterizing and tracking the infectious source of viral outbreaks. Therefore, NGS preparation should be performed with precautions, including periodic cleaning of laboratory equipment such as biosafety cabinets and pipettes, to avoid contamination and to obtain intact viral genome sequences because of low concentration of the pathogen in clinical samples.

## Active Targeted Surveillance Integrated by Epidemiological Surveys And Rodent Trapping

Surveillance is a critical component of prevention and control of infectious diseases (Langmuir, 1963). Epidemiological data are aggregated using an appropriate national institute for public health and environment in most countries where orthohantavirus infections have been diagnosed (Heyman et al., 2007). Passive surveillance is the most common method that relies on healthcare cooperation. It entails the regular collection of surveillance data and passive notifications that are generated and sent by local clinic or hospital physicians. However, passive surveillance has the following limitations: 1) physicians infrequently inform of cases, 2) physicians are unaware of reportable diseases, and 3) physicians are biased in reporting disease cases (Woolhouse and Gowtage-Sequeria, 2005; Jones et al., 2008; Levinson et al., 2013). In addition, identifying the infectious origin and responding to outbreaks rapidly is difficult when a newly emerging pathogen appears.

Active surveillance occurs when a health department is proactive and contacts health care providers or laboratories requesting disease information. Though this strategy involves additional cost and time, it tends to provide complete disease frequency estimation. This surveillance strategy actively searches for patients whose clinical characteristics are similar to viral symptoms. Public health centers and clinics, private clinics, and hospitals in endemic areas are the institutes that regularly collect information from responsive groups. In 2017 and 2018, active surveillance investigated the transmission route of sylvatic ZIKV in humans in ZIKV-endemic areas (Pauvolid-Correa et al., 2019). Active surveillance for ZIKV revealed that maximum mammal population in the active human transmission area was exposed to the virus in urban and peri-urban habitats of Brazil. In 2020, Dutch public health services collected the samples from the suspected cases of the infectious coronavirus disease 2019 from physicians and laboratories for three weeks (Munnink et al., 2020). Using epidemiological information obtained from the patients and whole-genome sequencing, they investigated the genetic diversity of the multiple introduction events and transmission patterns in the Netherlands. This active surveillance controlled the spreading of Severe Acute Respiratory Syndrome Coronavirus 2 among the public and developed social distancing strategies and policies to prevent further spread.

In Germany and Finland, active surveillance by studying the spatial and temporal dynamics of zoonotic pathogens and monitoring reservoir host population and clinical HFRS cases contributed to prediction and prevention of rodent-borne hantavirus infections (Korpela et al., 2013; Voutilainen et al., 2016; Vanwambeke et al., 2019). The precise infection site could not be identified using active surveillance; however, intensive and long-term datasets provided a solid basis for understanding PUUV infection dynamics in bank voles.

**Figure 2:**
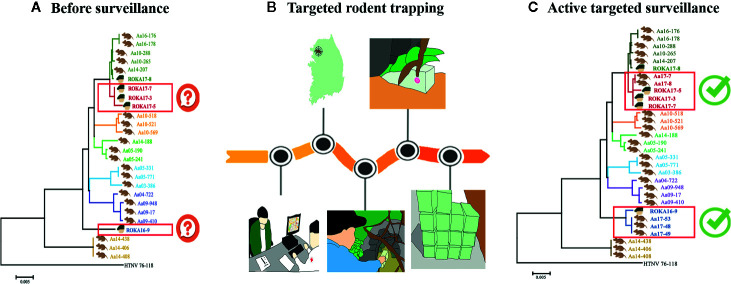
Elaborate and accurate improvement of tracking orthohantavirus using targeted rodent trapping. **(A)** Phylogenetic analyses before surveillance. The whole-genome sequences of orthohantavirus from patients with hemorrhagic fever with renal syndrome (HFRS) did not cluster with a specific location. **(B)** Epidemiological surveys and active targeted rodent trapping in the suspected area. The epidemiological interviews were performed for precise virus tracking. Active targeted trapping was performed in the place predicted to be infected. **(C)** Phylogenetic analyses after active targeted surveillance. The whole-genome sequences of orthohantavirus from rodent samples trapped in the suspected site were clustered with the orthohantavirus from patients with HFRS.

In the ROK Army (ROKA), active surveillance was implemented by the Armed Forces Capital Hospital to track HFRS outbreaks in military personnel (Song et al., 2006). The potential transmission events and risk factors associated with the viral infection were determined by conducting epidemiological interviews **(** **Table 2** **)**. The questionnaire must obtain a specific set of metadata to unveil the suspected infection source: 1) travels, exercises, and outdoor activities; 2) clinical complication progression; 3) contact time of suspected sources prior to the onset date; and 4) vaccination. As interviewees might not remember all the information, we found that the suspected site and timing of infection should be considered carefully while conducting epidemiological interviews and records. Based on the epidemiological interviews, a rapid responsive team collected rodents in military training areas near the putative sites and analyzed the samples for viral genome sequences using RT-PCR and NGS. The HTNV genome sequences obtained from the rodents collected at the suspected site were phylogeographically analyzed with the genome sequences obtained from the samples of patients with HFRS. Phylogeographic analyses showed that four ROKA patients with HFRS from different military bases participated in off-site military exercises within 30 days prior to the onset of clinical symptoms (Kim et al., 2019) **(** **Figure 2** **)**. This active targeted surveillance allowed for phylogenetic distinction (resolution) within a very short distance (approximately 5 km) to track the precise infection site.

**Table 2:** Questionnaires for patients suspected to have hemorrhagic fever with renal syndrome (HFRS).

1. Clinical symptoms (If you have any of the following symptoms)?	Yes or No
Fever	**Yes or No**
Headache	**Yes or No**
Nausea	**Yes or No**
Flank pain	**Yes or No**
Chest pain	**Yes or No**
Myalgia	**Yes or No**
Diarrhea	**Yes or No**
Sore throat	**Yes or No**
Dizziness	**Yes or No**
Dyspnea	**Yes or No**
Weakness	**Yes or No**
Hyperemia	**Yes or No**
Red spots on skin	**Yes or No**
Decreased urine output	**Yes or No**
Excessive urine volume	**Yes or No**
Hematuria	**Yes or No**
Others (hemodialysis, transfusion, administration of antibiotics, etc.)	**Yes or No**
**2.Vaccination**	
If yes, how many times vaccinated?	**( ) times or No**
**3. Epidemiologic Review**	
Was there animal contact within 30 days of disease onset?	**Yes or No**
If Yes, describe type, location, date, and nature of contact:	**Yes or No**
Answer based on activity between 10 and 30 days	
1) Was there field duty?	**Yes or No**
2) If Yes, Did soldier sleep on the ground?	**Yes or No**
3) Did soldier low crawl in the grass?	**Yes or No**
4) Sleep in tent?	**Yes or No**
5) Notice rat or mice droppings?	**Yes or No**
6) Use vegetation as camouflage?	**Yes or No**
7) See rodent?	**Yes or No**
8) Drive enclosed vehicle?	**Yes or No**
9) What other potential exposes are given?	**Yes or No**
10) Vacation or travel outside of the workplace?	**Yes or No**

## Preventive Strategies: Warning Signs, Rescheduling, Clearance, And Vaccination

Hantaviruses may be actively spread *via* rodents even in areas without patients with HFRS and through Orthohantavirus-contaminated rodent excreta. Preventive measures are needed in endemic areas identified by active surveillance to mitigate HFRS outbreaks. Endemic locations should be cleaned and rearranged to avoid contact with rodents. The following measures can be undertaken: 1) setting up a trash can with a cover, 2) cutting the grass on the ground, 3) spraying water to prevent dust prior to exercises or activities, and 4) installment of public warning signs to prevent human access to endemic areas (Kim et al., 2019). An aggressive preventive strategy can be established by rescheduling additional events or military exercises in these areas. Moreover, vaccination is remarkably important because of the lack of effective therapeutics against orthohantavirus infection (Schmaljohn, 2009). In the ROK, different HFRS severity profiles were observed between vaccinees and non-vaccinees against *Orthohantavirus*, demonstrating moderate vaccination effectiveness in endemic high-risk population (Jung et al., 2018; Yi et al., 2018). Thus, vaccinating the people living or working in endemic areas for orthohantaviruses is a pivotal strategy for disease prevention.

## Future Directions and Other Applications

Apart from NGS technologies, advanced bioinformatics techniques are required to investigate for genomic epidemiology. Recent studies have reported viral evolutionary, genomic, and epidemiological dynamics of influenza virus, ZIKV, and EBOV using Bayesian Evolutionary Analysis by Sampling Trees (BEAST) (Rambaut et al., 2008; Gire et al., 2014; Faria et al., 2016). However, the knowledge on the spatiotemporal epidemiology and genetic characteristics of hantavirus is limited. The BEAST provides insights into hantavirus evolutionary dynamics, epizootiologic surveys, and phylogeographic analyses. NGS techniques are rapid screening and whole-genome sequencing platforms enabling viral genome analyses, identification of emerging viruses, transmission chains, diagnostics, and therapeutics (Hoenen et al., 2016). The MinION system (Oxford Nanopore Technologies, Oxford, UK) is a portable sequencing device with weighing less than 100 g (Jain et al., 2015; Castro-Wallace et al., 2017; Goordial et al., 2017). Some studies have reported improved genome epidemiology of EBOV during outbreaks under resource-limited conditions using NGS (Quick et al., 2016; Naveca et al., 2019). Thus, advanced bioinformatics and real-time NGS of viral pathogens are of utmost importance for etiological agent diagnosis and point-of-care in the field (Greninger et al., 2015; Jain et al., 2016).

## Conclusion

**Figure 3:**
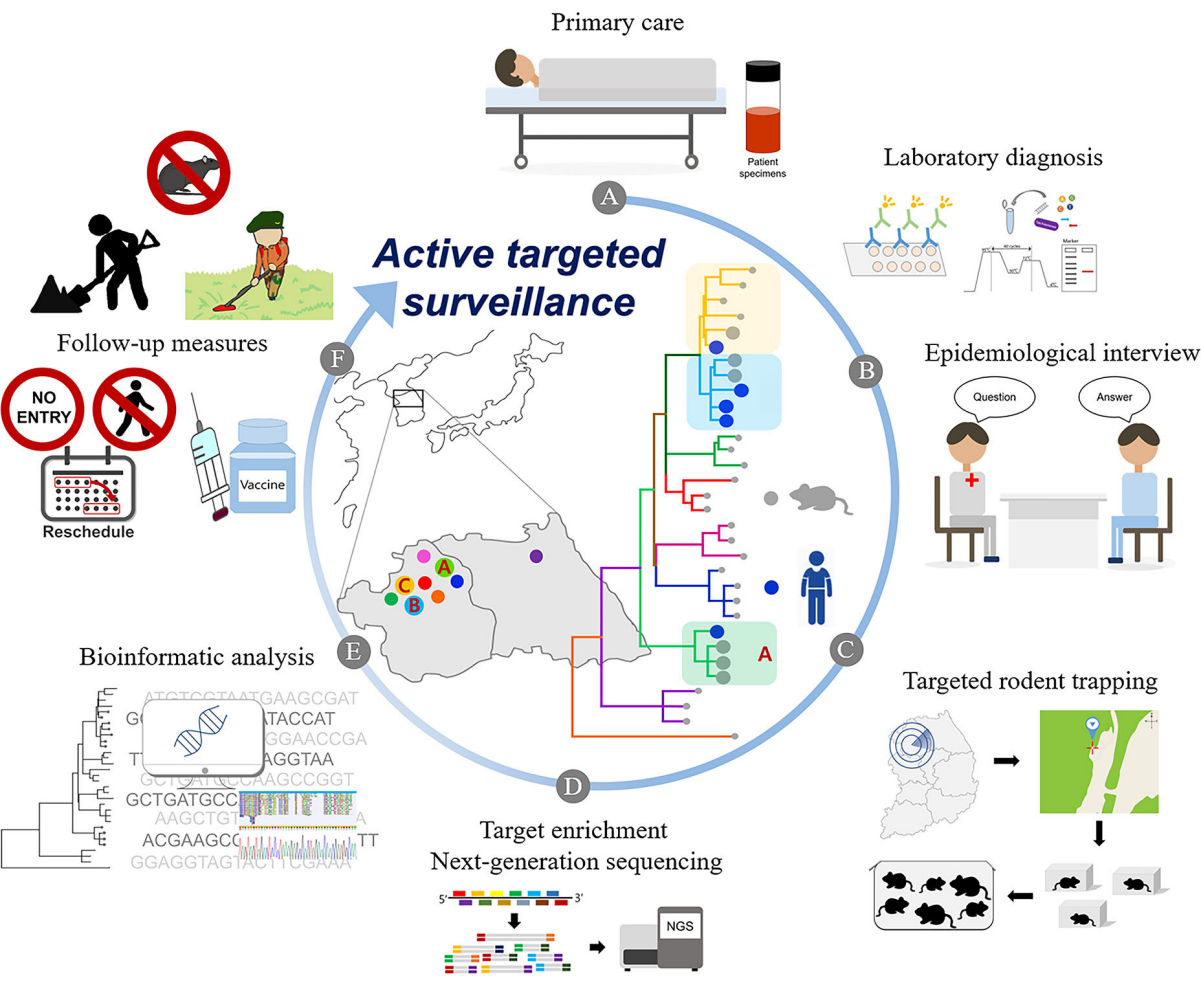
Active targeted surveillance for the identification of infection sites of emerging orthohantavirus outbreaks. An infection location of a patient with hemorrhagic fever with renal syndrome (HFRS) was identified using active targeted surveillance. **(A)** The patient is hospitalized with suspected HFRS. **(B)** Laboratory diagnosis confirms orthohantavirus infection using serological and molecular tests. The epidemiological interview is conducted to identify a suspected site of emerging orthohantavirus. **(C)** A targeted rodent trapping is performed at the suspected infection site. **(D)** Using a target enrichment NGS method, whole-genome orthohantavirus sequences are recovered from patients with HFRS and orthohantavirus-infected rodents. **(E)** Bioinformatic analyses provide high-resolution phylogeographic links between patient- and rodent-derived orthohantavirus strains. “Patient A” is highly suggestive to be infected with *orthohantavirus* circulating in the green area. **(F)** Active surveillance provides follow-up measures for mitigating HFRS incidences by cleaning the sites, placing warning signs, rescheduling activities, and vaccinating the high-risk population in the endemic areas.

In this study, we reviewed the active surveillance for emerging hantaviruses by focusing on concurrent genome sequencing, epidemiological data, and targeted rodent trapping (**Figure 3** and **Box 1**). We found that fresh sera of patients in the early phase of the disease should be obtained for diagnosing the infection by recovering whole-genome sequence of the virus with high coverage. Moreover, epidemiological interviews facilitate the identification of the infection time and the location where a patient was exposed to the infection. Viral genomic sequences from natural reservoirs will help in defining the infectious source and the site of hantavirus-induced diseases. Furthermore, a robust collaboration among physicians, epidemiologists, ecologists, microbiologists, molecular biologists, zoologists, immunologists, entomologists, and bioinformaticians will reinforce genomic epidemiology and active surveillance to develop an effective prevention strategy against emerging hantavirus outbreaks.

Box 1 **Hypothetical scenario: Active targeted surveillance through an outbreak of hemorrhagic fever with renal syndrome (HFRS) in suspected patients.**

A 57-year-old man visited the hospital in Yeoncheon-gun with continuous fever and dizziness. He told the doctor that he was a farmer who worked in a field near the Hantaan river and had harvested crops. There was point bleeding in the palate and conjunctival hemorrhage. He was hospitalized in the emergency room. His body temperature was normal, but his blood pressure was 85/55. The blood and urine test results showed high white blood cell counts (14,500) and low specific gravity of urine (1.015). He had hypotension and acute renal failure, and he received fluid therapy during admission in the emergency room (**Figure 3A**). HFRS was suspected, and the exact etiologic agent was analyzed by laboratory diagnostics. RT-PCR and indirect immunofluorescence assay showed that the causative factor was Hantaan virus (HTNV). An epidemiological interview was conducted to track a suspected emerging HTNV site. The patient said that he had worked in the field to harvest crops 15 days ago, and he was at home for the last 3 days. He also said that he had seen rodents while working, approximately 10 days ago (**Figure 3B**). For precise virus tracking, active targeted trapping was performed using a rapid responsive team in suspected field sites (**Figure 3C**). In total, 24 rodents were trapped over 3 days, of which four were HTNV-positive. Targeted sequencing (multiplex PCR-based) of samples from the patient and captured rodents revealed that the HTNV from the patient clustered on the phylogenetic tree and shared a geographical characteristic with the rodent-derived viruses (**Figures 3D, E**). The results confirmed the area where the patient was infected, and then the local health institute restricted field activities such as farming in the area. In addition, residents of the endemic area were encouraged to receive vaccinations, and environmental cleaning was improved to eliminate rodents (**Figure 3F**).

## Author Contributions

W-KK, SC, S-HL, JN, G-YL, and KP wrote and prepared the original draft. W-KK, DL, SJ, and J-WS wrote, reviewed, and edited the manuscript. J-WS supervised the study and acquired the funding. All authors contributed to the article and approved the submitted version.

## Funding

This work was supported by the Agency for Defense Development (UE202026GD) and the Research Program to Solve Social Issues of the National Research Foundation of Korea (NRF) funded by the Ministry of Science and ICT (NRF-2017M3A9E4061992).

## Conflict of Interest

The authors declare that the research was conducted in the absence of any commercial or financial relationships that could be construed as a potential conflict of interest.
